# The Utility of Transperineal Template Saturation Biopsy in the Detection of Clinically Significant Prostate Cancer

**DOI:** 10.1155/aiu/9961847

**Published:** 2025-03-05

**Authors:** Kenta Onishi, Yasushi Nakai, Tatsuki Miyamoto, Fumisato Maesaka, Mitsuru Tomizawa, Takuto Shimizu, Shunta Hori, Daisuke Gotoh, Makito Miyake, Tetsuya Tachiiri, Nagaaki Marugami, Kiyohide Fujimoto, Nobumichi Tanaka

**Affiliations:** ^1^Departments of Urology, Nara Medical University, Kashihara, Nara, Japan; ^2^Departments of Radiology and Nuclear Medicine, Nara Medical University, Kashihara, Nara, Japan; ^3^Departments of Prostate Brachytherapy, Nara Medical University, Kashihara, Nara, Japan

**Keywords:** clinically significant prostate cancer, multiparametric magnetic resonance imaging, transperineal template saturation biopsy

## Abstract

**Aim:** We investigated the role of transperineal template saturation biopsy (TTSB) in detecting clinically significant prostate cancer (csPCa). We compared the TTSB findings with multiparametric magnetic resonance imaging (mpMRI) findings in suspected prostate cancer patients.

**Methods:** This retrospective study included 124 patients who underwent TTSB following mpMRI at our institute. We examined factors contributing to csPCa detection in these patients. We examined the association between the Prostate Imaging-Reporting and Data System (PI-RADS) Version 2.1 category and csPCa detection.

**Results:** The median age at TTSB was 68 (interquartile range: 62–73) years, and the median prostate-specific antigen level was 9.9 (6.1–15.5) ng/mL. Herein, 61.3% (76/124) of the patients who underwent TTSB had cancer and 35.5% (44/124) had csPCa. Abnormal digital rectal examination findings (*p*=0.006) and PI-RADS category ≥ 4 (*p* < 0.001) were independent factors for csPCa detection. Among patients categorized as PI-RADS ≥ 4, 64.8% (35/54) had csPCa; csPCa frequency increased with increasing PI-RADS categories (*p* < 0.001). Cancer was detected in 38.3% (23/60) of the patients categorized as PI-RADS ≤ 2; among them, 10% (6) had csPCa. Only 3.2% (4/124) of the patients had TTSB-related adverse events ≥ grade 2, 0.8% (1/124) suffered from hematuria, and 2.4% (3/124) had acute urinary retention. All patients were treated conservatively.

**Conclusions:** Patients with a higher PI-RADS category tended to have csPCa. However, the PI-RADS category alone may not be sufficient for csPCa detection. TTSB detected csPCa in 10% of the patients with negative mpMRI findings. TTSB is a safe and crucial technique for accurately diagnosing prostate cancer.

## 1. Introduction

Prostate cancer is one of the leading causes of death in men, the second most common cancer, and the fifth leading cause of mortality worldwide [1]. The clinical features of prostate cancer range from nonlethal indolent cancers to fatal advanced cancers [2]. Previously, the primary procedure for prostate cancer diagnosis was transrectal ultrasound (TRUS)-guided systematic biopsy based on prostate-specific antigen (PSA) levels and digital rectal examination (DRE) findings. We previously examined the optimal number of cores for prostate biopsy using nomograms based on age and prostate volume [3]. However, this procedure alone has the limitation of overdiagnosing clinically insignificant prostate cancer (ciPCa) that does not require treatment and underdiagnosing clinically significant prostate cancer (csPCa) that requires treatment [4, 5]. Recent advances in multiparametric magnetic resonance imaging (mpMRI) have made it possible to accurately differentiate csPCa from ciPCa and benign lesions with high accuracy [6]. With the development of mpMRI, fusion-guided targeted biopsy has become popular, and a high cancer detection rate has been reported [4, 7, 8]. However, although the utility of mpMRI has been reported, some prostate cancer lesions cannot be detected using this procedure alone. Furthermore, despite improvements in the diagnostic ability of fusion-guided targeted biopsy, using it alone may make physicians overlook high-risk prostate cancer [9–11].

To date, investigators have used the findings of prostatectomy specimens for comparison in studies evaluating the diagnostic ability of mpMRI [12, 13]. However, comparisons with whole prostate specimens are not ideal due to selection bias, as only patients with prostate cancer eligible for curative treatment are included. In contrast, transperineal template saturation biopsy (TTSB) can be used to obtain tissue from the entire prostate gland and can be used to make accurate diagnoses without mpMRI results [12, 14]. Thus, selection bias is unlikely to occur when using TTSB findings for evaluating the diagnostic ability of mpMRI.

Herein, we compared mpMRI findings with TTSB pathology findings in patients who underwent subsequent mpMRI and TTSB.

## 2. Materials and Methods

### 2.1. Patient Population

This retrospective study initially comprised 522 patients who underwent TTSB at Nara Medical University Hospital between November 2005 and March 2022. Patients who did not undergo mpMRI before TTSB and those who underwent cognitive biopsy or fusion-guided targeted biopsy combined with TTSB were excluded; finally, 124 patients were included in the analyses.

### 2.2. Procedure of TTSB

**Figure 1:**
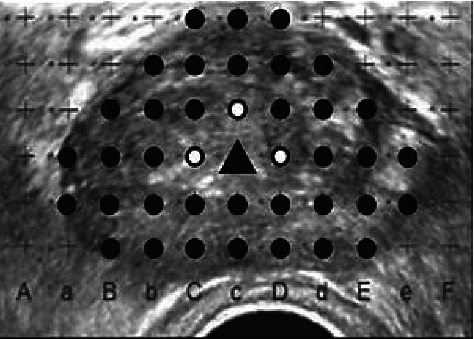
Transrectal ultrasound image at transperineal template saturation biopsy. Ultrasound image of the prostate showing the sites for sampling (black dots). Triangle shows urethra. White dots show sites where sampling was avoided.

TTSB was performed in the dorsal lithotomy position under either general or spinal anesthesia, and a 14-French urethral catheter was inserted prior to the procedure. All patients received oral premedication with a single intravenous dose of cefazolin (1 g) or levofloxacin (500 mg) to prevent TTSB-related infections. A transrectal probe (Toshiba Medical, Tochigi, Japan, or ARIETTA 60, Hitachi, Tokyo, Japan) attached to a brachytherapy stepping unit (AccuSeed, Bedfordshire, UK, or Stepper for all ER probes; Medical Targeting Technologies GmbH, Lüneburg, Germany) was inserted into the rectum. Prostate volume was calculated using the following formula: length × width × height × 0.5236 [15]. The number of biopsy cores was estimated using the widest transverse section. The interval between biopsy cores in a row was uniformly 5 mm from the right to the left in the longitudinal view, except for the area nearest to and around the urethra (Figure 1). We performed a single longitudinal biopsy if the distance from the prostate apex to the base was less than 2 cm, whereas we performed an additional longitudinal biopsy if the distance was more than 2 cm. Eventually, the number of additional cores was determined using the calculated prostate volume, and the number of biopsy cores was determined based on the prostate volume. To achieve “saturation biopsy,” one biopsy core per 1 mL of prostate volume was required. The biopsy was performed using an 18 gauge, 25 cm long biopsy gun (Bard, Covington, GA, USA) [16].

### 2.3. Evaluation of Biopsy Findings for Cancer Detection

CiPCa using TTSB was defined by Epstein et al. [15] as follows: (a) Gleason score < 7 and the number of positive cores ≤ 3 or (b) Gleason score < 7 and the maximal coverage of cancer in one core < 4.5 mm, with the total coverage of cancer for all cores < 5.5 mm. csPCa was defined as cancer detected by TTSB, excluding ciPCa.

**Figure 2:**
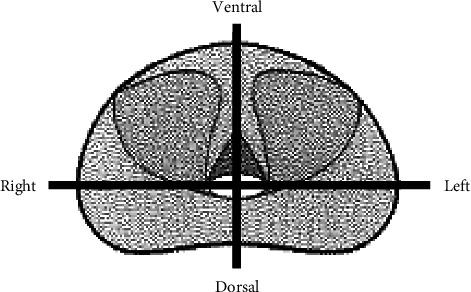
Schema of the divided prostate specimens. We divided the prostate into four regions to examine the concordance between the multiparametric magnetic resonance imaging findings and the pathological findings of the transperineal template saturation biopsy and prostatectomy specimens.

First, we examined the factors contributing to csPCa detection in 124 patients who underwent TTSB. Second, two radiologists (NM and TT) with expertise in urological imaging reviewed the images of 124 patients who underwent mpMRI prior to TTSB. They evaluated prostate lesions using the Prostate Imaging-Reporting and Data System (PI-RADS) Version 2.1 category and examined the relationship between the PI-RADS category and csPCa detection. Third, we examined the concordance of the MRI findings with the pathological findings of the TTSB and prostatectomy specimens in 14 patients who underwent radical prostatectomy after cancer detection via TTSB. We divided each prostate specimen into four sections (Figure 2) and examined the concordance of the MRI findings in 56 sections with the pathological findings of the TTSB and prostatectomy specimens. Finally, we evaluated the adverse event criteria using the Common Terminology Criteria for Adverse Events v 5.0.

### 2.4. Statistical Analysis

The Kruskal–Wallis and the chi-squared tests were used for the comparison of continuous and categorical variables, respectively, among the three groups. Binary logistic regression analysis was used to estimate the independent parameters for csPCa detection using TTSB. The cutoff value was determined as the point closest to the upper left-hand corner of the receiver operating characteristic curve. Variables were selected for the multivariable analysis if their *p* values were < 0.05 in the univariable analysis. The chi-squared test for trends was used for categorical variables. Statistical significance was set at *p* < 0.05. All statistical analyses were performed using PASW Statistics 17.0 (SPSS, Chicago, IL, USA) and Prism software 5.00 (GraphPad Software, San Diego, CA, USA).

## 3. Results

### 3.1. Patient Population

The median age at TTSB was 68 (interquartile range: 62–73) years, and the median PSA level was 9.9 (6.1–15.5) ng/mL. The median prostate volume and number of cores obtained using TTSB were 36.5 (29.7–48.5) mL and 35.5 (30–42), respectively. The median PSA density was 0.27 (0.16–0.43) ng/mL/mL. Herein, 53 (42.7%) were first biopsies, 41 (33.1%) were second biopsies, and 30 (24.2%) were third or later biopsies. Of the 124 patients, 82 (66.1%) showed abnormal mpMRI findings and 19 (15.3%) had abnormal DRE findings. Cancer was detected in 76 (61.3%) of 124 patients who underwent TTSB and 44 (35.5%) had csPCa (Table 1). In the comparison of background factors among the csPCa, ciPCa, and no cancer groups, significant differences were found in age at TTSB (*p*=0.002), PSA at TTSB (*p*=0.002), prostate volume (*p* < 0.001), the number of cores obtained using TTSB (*p*=0.001), PSA density (*p* < 0.001), and DRE findings (*p* < 0.001). Significant differences were found in neither the number of cores/prostate volumes (*p*=0.077) nor that of previous biopsies (*p*=0.76).

### 3.2. Factors for csPCa Detection

In the univariable analysis, a higher PSA (≥ 10 ng/mL) (*p*=0.005), a lower number of cores obtained from TTSB (≤ 34) (*p*=0.006), a higher PSA density (≥ 0.25 ng/mL/mL) (*p*=0.002), abnormal DRE findings (*p* < 0.001), and a higher PI-RADS category (≥ 4) (*p* < 0.001) were significant factors for csPCa detection. In the multivariable analysis, abnormal DRE findings (*p*=0.006) and a higher PI-RADS category (≥ 4) (*p* < 0.001) were independent factors for csPCa detection (Table 2). In fact, 86.7% (13/15) of the patients with PI-RADS category ≥ 4 and palpable tumors were diagnosed with csPCa. Meanwhile, only 7.1% (4/56) of the patients with PI-RADS category ≤ 2 and without palpable tumors were diagnosed with csPCa.

### 3.3. Adverse Events Associated With TTSB

TTSB-related grade 2 or higher adverse events were observed in four (3.2%) patients. One (0.8%) patient experienced hematuria that improved with the use of hemostatic agents for 3 days. Three (2.4%) patients experienced acute urinary retention and required catheterization for a temporary period; however, the catheters could be successfully removed after a median of 1 (range: 1–3) weeks with the administration of an alpha-1 adrenergic receptor antagonist. The median prostate volume was 66.6 (range: 46.8–84) mL.

### 3.4. Correlation Between mpMRI Findings and csPCa Detection

**Figure 3:**
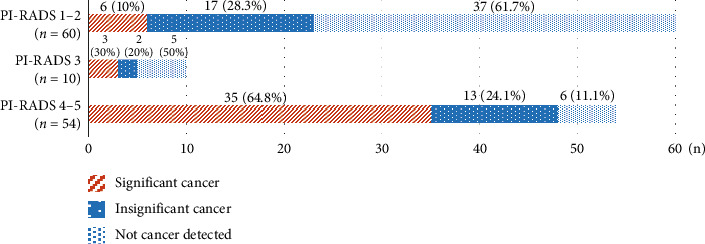
Cancer detection rate according to the prostate imaging-reporting and data system (PI-RADS) category (*n* = 124). Of the 124 patients, 60 (48.4%) were classified as prostate imaging-reporting and data system (PI-RADS) category 1-2, 10 (8.1%) as PI-RADS category 3, and 54 (43.5%) as PI-RADS category 4-5. Among the 54 patients classified as PI-RADS category 4-5, 35 (64.8%) were diagnosed with clinically significant prostate cancer. However, prostate cancer was not detected in 37 (61.7%) of the 60 patients classified as PI-RADS category 1-2; however, among them, six (10%) patients were diagnosed with clinically significant prostate cancer. PI-RADS, prostate imaging reporting and data system.

Of the 124 patients, 44 (35.5%) had csPCa, 32 (25.8%) had ciPCa, and 48 (38.7%) did not have prostate cancer. Of all patients, 54 (43.5%) were classified as PI-RADS ≥ 4, 10 (8.1%) as PI-RADS 3, and 60 (48.4%) as PI-RADS 1–2. A total of 35 (64.8%) patients classified as PI-RADS ≥ 4 were diagnosed with csPCa. Prostate cancer was not detected in 36 (67.9%) of the 53 patients classified as PI-RADS 1; however, four (7.5%) patients were diagnosed with csPCa (Figure 3). The incidence of csPCa increased with increasing PI-RADS category (*p* < 0.001) (Table 3). csPCa was observed in six (10%) patients with negative mpMRI findings (PI-RADS ≤ 2, *n* = 60, 48.4%).

### 3.5. Correlation Between mpMRI and Pathological Findings

The pathological findings of the prostatectomy specimens revealed cancers in 35 of 56 sections (62.5%); TTSB detected cancers in 28 of these 35 sections (80%). In particular, 12 (36.4%) of 33 sections identified by mpMRI as having no lesion were found to have cancer, of which 8 (66.7%) were detected by TTSB (Table 4(a)). Gleason Grade Group ≥ 2 cancers were found in 31 of 56 (55.4%) sections based on the pathological findings from prostatectomy specimens, of which 20 (64.5%) were detected by TTSB (Table 4(b)). Regarding the Gleason score of the prostatectomy specimens, only 2 of 14 cases (14.3%) among the TTSB specimens required upgrading; 12 cases (85.7%) were matched or required downgrading (Table 4(c)).

## 4. Discussion

Several studies have reported the high capacity of TTSB for prostate cancer detection [11, 16–18]. However, there are few reports on the factors influencing csPCa detection using TTSB. Kaufmann et al. reported that the significant predictors of csPCa in TTSB were increased PSA levels, an older age, a higher PI-RADS category, and a smaller prostate volume [14]. Herein, we demonstrated that abnormal DRE findings and a higher PI-RADS category were independent factors for csPCa detection in a population that included both initial and repeat biopsies. These results indicated the importance of mpMRI in detecting csPCa using TTSB. Recently, mpMRI with PI-RADS categorization has become a common tool for prostate cancer diagnosis [8, 19]. The usefulness of fusion-guided targeted biopsy for detecting csPCa has been reported [4, 20]. However, in 1.3%–16% of the cases, no lesions were detected on mpMRI, but csPCa was observed in systematic biopsies [21–26]. In addition, Gündoğdu et al. compared the pathological findings of prostatectomy specimens with mpMRI findings and concluded that the PI-RADS category alone is inadequate for csPCa detection [27]. In addition, fusion-guided targeted biopsy alone has been reported to overlook 19.9%–27% of the csPCa cases in prostate cancer diagnosis [14, 28]. Sonmez et al. reported that the ideal number of cores to be obtained from each suspicious lesion in targeted biopsy depends on the characteristics of the lesions, and although obtaining two or three biopsy cores may be adequate in PI-RADS 4 and 5 lesions, a minimum of four biopsy cores should be obtained from PI-RADS 3 lesions to ensure accurate histopathological results [29]. In this study, csPCa was identified in 6 (10%) out of 60 cases with negative mpMRI findings (PI-RADS category ≤ 2). The PI-RADS category alone may not be adequate for the detection of csPCa, and TTSB remains an indispensable method for the accurate diagnosis of prostate cancer.

TTSB is a useful diagnostic procedure. The transperineal approach has a lower incidence of sepsis after biopsy than the transrectal approach [30]. In addition, transperineal biopsy improves the detection of csPCa located in the anterior zone of the prostate, especially in patients who have undergone repeated biopsies or are enrolled in active surveillance protocols [31, 32]. TTSB is also suitable for indicating focal therapy because of its accurate diagnosis. Currently, we perform focal low-dose-rate brachytherapy for patients with low Gleason scores and low positive core rates in the unilateral lobe based on TTSB mapping. However, TTSB is limited by certain complications, especially acute urinary retention, which can be problematic. The incidence of acute urinary retention after TTSB has been reported to be 10%–11.5% [33, 34]. Herein, we observed a low acute urinary retention rate (2.4%). A possible reason for this may be the smaller prostate volume in the patient population compared with that in other reports of saturation biopsies. Overnight catheterization in all patients and avoiding needle insertion into the periurethral region may be another reason.

This study has several limitations. First, it was a retrospective study. Second, this was a small-scale cohort study. In particular, the number of patients who underwent prostatectomy after MRI and TTSB was small. Although TTSB can be used to obtain tissue from all parts of the entire prostate, more cases are needed to demonstrate the correlation of mpMRI with pathological findings in TTSB and prostatectomy specimens. Third, the present study classifies significant cancer using the definition of significant cancer in saturation biopsy published by Epstein et al. in 2005 [15]. This criterion is about 20 years old and may differ from actual clinical practice today. We believe that the definition of significant cancer in saturation biopsy needs to be revised. Fourth, the present study included patients who had previously undergone prostate biopsy. Fifth, we perform TTSB to avoid overtreatment and missing significant cancers. However, it is possible that we are detecting insignificant cancers, resulting in overdiagnosis. Factors that help avoid unnecessary biopsies are difficult to identify from the results of the present study. Based on the results of this multivariable analysis, the detection rate of significant cancer was as low as 7.1% (4/56) for patients with PI-RADS category ≤ 2 and without palpable tumor. However, we believe that it is necessary to create a nomogram that considers those two factors as well as other factors such as age and PSA density. Finally, we were unable to compare the detection abilities of TTSB and fusion-guided targeted biopsy using mpMRI. Recently, the utility of ^68^Ga-PSMA targeted biopsy for the detection of csPca has been reported [35]. In the future, we would like to accumulate more targeted biopsy cases to examine whether targeted biopsy or TTSB is more appropriate for csPCa detection.

## 5. Conclusions

Abnormal DRE findings and higher PI-RADS scores were independent predictors of csPCa detection using TTSB. csPCa was detected in patients with higher PI-RADS categories. However, there were a few cases in which csPCa could not be detected using mpMRI. We believe that TTSB remains essential for the accurate diagnosis of prostate cancer.

## Figures and Tables

**Table 1:** Patient characteristics (*n* = 124).

Variables		*n* = 124	csPca	ciPca	No cancer	*p* value
			*n* = 44	*n* = 32	*n* = 48	
Median age at TTSB, years (IQR)		68 (62–73)	69 (63–73)	70.5 (66–74)	65.5 (60–70)	0.002^†^

Median PSA at TTSB, ng/mL (IQR)		9.88 (6.07–15.5)	13.95 (6.69–23.2)	7.54 (5.89–11.3)	9.32 (6–15.6)	0.002^†^

Median prostate volume, mL (IQR)		36.5 (29.7–48.5)	34 (25.2–41.8)	31.5 (25.5–40.7)	44.8 (35.8–54.6)	< 0.001^†^

Median number of cores obtained from TTSB (IQR)		35.5 (30–42)	33 (28–38)	33.5 (28–40.3)	40 (34.8–47)	0.001^†^

Median number of cores/prostate volume, /mL (IQR)		1.03 (0.91–1.23)	1.05 (0.93–1.27)	0.95 (0.86–1.07)	1.05 (0.94–1.28)	0.077^†^

Median PSA density, ng/ml/ml (IQR)		0.27 (0.16–0.43)	0.4 (0.24–0.62)	0.21 (0.14–0.35)	0.21 (0.13–0.35)	< 0.001^†^

Number of previous biopsies (%)	0	53 (42.7%)	21 (47.7%)	14 (43.8%)	18 (37.5%)	0.76^‡^
	1	41 (33.1%)	12 (27.3%)	12 (37.5%)	17 (35.4%)	
	2 <	30 (24.2%)	11 (25%)	6 (18.8%)	13 (27.1%)	

DRE, findings (%)	Normal	105 (84.7%)	29 (65.9%)	30 (93.8%)	46 (95.8%)	< 0.001^‡^
	Abnormal	19 (15.3%)	15 (34.1%)	2 (6.3%)	2 (4.2%)	

Abbreviations: ciPCa, clinically insignificant prostate cancer; csPCa, clinically significant prostate cancer; DRE, digital rectal exam; IQR, interquartile range; MRI, magnetic resonance image; PSA, prostate-specific antigen; TTSB, transperineal template saturation biopsy.

^†^Kruskal–Wallis test.

^‡^Chi-square test.

**Table 2:** Univariable and multivariable logistics regression analysis results for significant cancer detection by transperineal template saturation biopsy (*n* = 124).

Factors		Univariable analysis			Multivariable analysis		
		Odds ratio	95% CI	*p* value	Odds ratio	95% CI	*p* value
Age at TTSB, years	≤ 69	1					
	≥ 70	1.3	0.62–2.73	0.49			

PSA at TTSB, ng/mL	< 10	1			1		
	≥ 10	3.1	1.4–6.87	0.005	2.41	0.68–8.55	0.17

Number of cores obtained from TTSB	≤ 34	1			1		
	≥ 35	0.34	0.16–0.74	0.006	0.62	0.22–1.72	0.36

PSA density, ng/ml/ml	< 0.25	1			1		
	≥ 0.25	3.43	1.55–7.61	0.002	1.5	0.42–5.33	0.53

Biopsy	Intial	1					
	Repeat	0.73	0.35–1.53	0.41			

CRP, mg/dL	> 0.1	1					
	≤ 0.1	1.37	0.65–2.89	0.4			

DRE, findings	Normal	1			1		
	Abnormal	9.83	3.01–32.1	< 0.001	7.21	1.76–29.5	0.006^#^

PI-RADS category	≤ 3	1			1		
	≥ 4	11.7	4.79–28.4	< 0.001	7.32	2.76–19.4	< 0.001^#^

Abbreviations: CI, confidence interval; CRP, c-reactive protein; DRE, digital rectal exam; PI-RADS, prostate imaging-reporting and data system; PSA, prostate specific antigen; TTSB, transperineal template saturation biopsy.

^#^Binomial logistic regression analysis.

**Table 3:** Diagnostic accuracy for significant cancer detection by PI-RADS category and transperineal template saturation biopsy.

	PI-RADS 1	PI-RADS 2	PI-RADS 3	PI-RADS 4	PI-RADS 5	*p* value
Significant cancer (+) (*n* = 44)	4 (9.1%)	2 (4.5%)	3 (6.8%)	15 (34.1%)	20 (45.5%)	*p* < 0.001^∗^
Significant cancer (−) (*n* = 80)	49 (61.3%)	5 (6.3%)	7 (8.8%)	10 (12.5%)	9 (11.3%)	

⁣^∗^Chi-square test for trend.

Abbreviation: PI-RADS, prostate imaging reporting and data system.

**Table 4:** Diagnostic accuracy for the detection of (a) any cancer and (b) grade group ≥ 2 cancer between pathological findings of the prostatectomy and TTSB specimens (*n* = 56) and (c) downgrading and upgrading of gleason scores between TTSB and radical prostatectomy (*n* = 14).

**(a)**			

**MRI findings**	**TTSB**	**Prostate specimen**	**Detection rate by TTSB (%)**
Total (*n*-56)	28 (50%)	35 (62.5%)	80
PI-RADS 1–2 (*n* = 33)	8 (24.2%)	12 (36.4%)	66.7
PI-RADS 3 (*n* = 5)	4 (80%)	5 (100%)	80
PI-RADS 4–5 (*n* = 18)	16 (88.9%)	18 (100%)	88.9

**(b)**			

**MRI findings**	**TTSB**	**Prostate specimen**	**Detection rate by TTSB (%)**
Total (*n*-56)	20 (35.7%)	31 (55.4%)	64.5
PI-RADS 1–2 (*n* = 33)	3 (9.1%)	11 (33.3%)	27.3
PI-RADS 3 (*n* = 5)	2 (40%)	3 (60%)	66.7
PI-RADS 4–5 (*n* = 18)	15 (83.3%)	17 (94.4%)	88.2

**(c)**			

	***n* = 14**		
Upgrading	2 (14.3%)		
Matching	10 (71.4%)		
Downgrading	2 (14.3%)		

Abbreviations: MRI, magnetic resonance image; PI-RADS, prostate imaging-reporting and data system; TTSB, transperineal template saturation biopsy.

## Data Availability

The datasets generated and/or analyzed during the current study are not publicly available to protect study participant privacy but are available from the corresponding author upon reasonable request.
